# Hybrid Integration of Magnetoresistive Sensors with MEMS as a Strategy to Detect Ultra-Low Magnetic Fields

**DOI:** 10.3390/mi7050088

**Published:** 2016-05-11

**Authors:** João Valadeiro, Susana Cardoso, Rita Macedo, Andre Guedes, João Gaspar, Paulo P. Freitas

**Affiliations:** 1 Instituto de Engenharia de Sistemas de Computadores-Microsystems and Nanotechnology (INESC-MN), Rua Alves Redol, No. 9, Lisboa 1000-029, Portugal; scardoso@inesc-mn.pt (S.C.); pfreitas@inesc-mn.pt (P.P.F.); 2 Instituto Superior Técnico IST, Physics Department, Universidade de Lisboa, Lisbon 1049-001, Portugal; 3 Picosense, Inc., 1900 Addison St Ste 200, Berkeley, CA 94704, USA; ritamacedo@gmail.com (R.M.); andre.aires@gmail.com (A.G.); 4 International Iberian Nanotechnology Laboratory (INL), Av. Mestre Jose Veiga, Braga 4715-330, Portugal; Joao.Gaspar@inl.int

**Keywords:** magnetoresistive sensors, noise, MEMS, hybrid devices, frequency modulation, magnetic field detection

## Abstract

In this paper, we describe how magnetoresistive sensors can be integrated with microelectromechanical systems (MEMS) devices enabling the mechanical modulation of DC or low frequency external magnetic fields to high frequencies using MEMS structures incorporating magnetic flux guides. In such a hybrid architecture, lower detectivities are expected when compared with those obtained for individual sensors. This particularity results from the change of sensor’s operating point to frequencies above the 1/*f* noise knee.

## 1. Introduction

The detection of low-intensity magnetic fields (down to pico-Tesla range) plays a major role in pushing the limits and widen the use of magnetic sensors in industrial applications [1,2] from automotive control to non-destructive tests (NDT) [3], being pivotal in the biomedical field, where it stands out in the bio-molecular recognition [4] and bio-signals imaging [5]. Both magnetic field sensitivity and intrinsic noise level set the sensor’s minimum field detected, being the latter the focus of this review. Presently, magnetic field sensing technologies include: superconducting quantum interference devices (SQUIDs)—the uppermost technique used for neuroimaging; fluxgate magnetometers [6]—prevalent in military industry (e.g., aircraft compass heading systems); Hall effect sensors [7]—widely use in low cost position sensor applications and Magnetoresistive (MR) sensors [8]—traditionally integrated in data storage industry [9].

The ability to operate at room temperature combined with (i) a high signal to noise ratio (SNR) in a large bandwidth (from DC to hundreds of MHz); (ii) a small footprint (μm scale lithographed areas) and (iii) low power consumption (~mW) allowed MR sensors to suit a wide range of applications beyond data storage, being a reliable alternative for detection at pico to mili-Tesla range. Detection limits below pT/Hz^1/2^ have already been demonstrated using MR sensors based on MgO magnetic tunnel junctions (MTJs) [10]. However, in the low frequency range the intrinsic noise of MR sensors is dominated by the 1/*f* magnetic component, which limits the minimum detectable field; at high frequency the sensor noise is reduced to the thermal level.

The use of a large array of small sensing elements connected in series [11] or patterning single sensing elements with larger active areas [12] are among the different strategies used to reduce the noise of MR sensors. On the other hand, instead of acting in the sensor intrinsic noise, a reliable alternative consists in the inclusion of AC microelectromechanical systems (MEMS) resonators with incorporated magnetic flux concentrators (MFCs) to modulate (quasi-)DC signals to an operating frequency (above the 1/*f* knee) where the thermal-mechanical noise dominates, and the total noise is minimum.

This review focuses on the hybrid integration of MR sensors with MEMS in an attempt to enhance the detection level of the device, exploiting the operation details and advantages of AC modulation at high frequency. This strategy allows magnetic detection of low-intensity and low-frequency signals when compared with conventional techniques centered on the reduction of the sensors intrinsic noise at low frequency.

## 2. Background

### 2.1. Magnetoresistive Sensors

**Figure 1:**
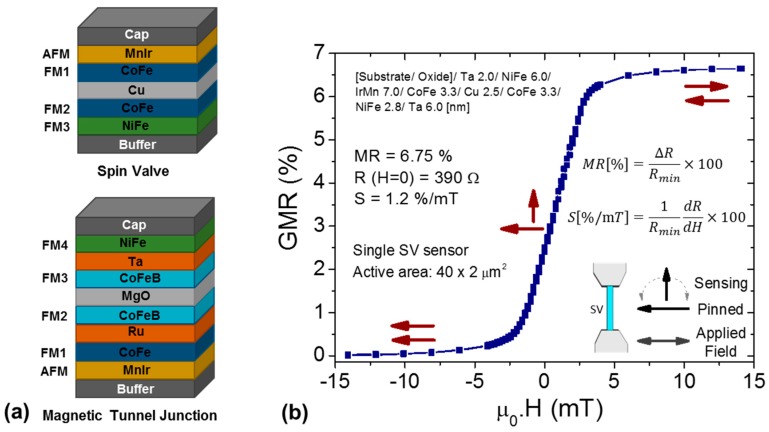
(**a**) Schematic view of a typical multilayer for spin-valves (SV) and magnetic tunnel junction (MTJ) sensors. SV: antiferromagnet (AFM), pinned layer (FM1), conductive spacer (Cu), sensing layer (FM2 and FM3). MTJ: antiferromagnet (AFM), pinned layer (FM1), insulator spacer (MgO), sensing layer (FM3 and FM4); a synthetic antiferromagnet (SAF) is obtained with the tri-layer FM1/Ru/FM2. The pinned layer magnetization is set in a fixed direction due to exchange-bias at AFM/FM interface; (**b**) Representative magnetotransport curve obtained for a patterned SV sensor (active area: 40 × 2 µm^2^) exhibiting a linear, centered and hysteresis free response. Red arrows illustrate the relative orientation of the in-plane magnetization in both pinned and sensing layers. *S* stands for the sensor sensitivity.

The resistance of a MR sensor changes with the variation of the external magnetic field, being the magnetoresistance value defined as the total resistance variation (Δ*R*) normalized to its minimum value (*R_min_*). Ferromagnetic/non-magnetic heterostructures such as spin-valves (SV) and magnetic tunnel junctions (MTJ) offer advantages over the single ferromagnetic films exhibiting (anisotropic magnetoresistance (AMR)), due to the magnetic stability and larger output signals. Either types of sensing devices are composed by a four layer structure: two ferromagnets (FM) separated by a non-magnetic layer (conductor in SVs; insulator in MTJs) and an antiferromagnet (AFM) responsible for setting the magnetization of a FM layer in a fixed direction (reference) (Figure 1a). In a SV sensor [13] the current flows in plane (CIP) and the resistance variation arises from spin dependent scattering of conduction electrons. In a MTJ, the electrons have to tunnel across the insulating barrier (<1 nm thick) as a result of a spin dependent tunneling probability, which originates an electron flow perpendicular to the plane (CPP). While the former display a magnetoresistance around 5%–10%—typical structure: Ni_80_Fe_20_/Co_80_Fe_20_/Cu/Co_80_Fe_20_/Mn_76_Ir_24_ (Figure 1b), the latter are characterized by a larger resistance variation, reaching tunnel magnetoresistance values at room temperature of 50%–70% in structures with amorphous AlO*x* barriers (incoherent electron tunneling) [14] or over 200% upon annealing with crystalline MgO barriers (coherent electron tunneling)-typical structure: Ir_20_Mn_80_/Co_70_Fe_30_/Ru/Co_40_Fe_40_B_20_/MgO/Co_40_Fe_40_B_20_/Ta/Ni_80_Fe_20_ [15]. The signal level achieved is proportional to the magnetoresistance ratio [16], therefore crystalline MTJ devices are pointed as the most promising candidates for a high sensitive field sensor. The sensitivity of a MgO-based MTJ is typically tens of %/mT, while for a SV sensor is ~1%/mT. However, extra noise sources, materials and annealing treatments required for a linear response [17,18] are additional issues to be considered in MTJs, when compared to AMR or SV devices. A fair comparison should take into consideration noise levels, to better address the minimum detectable fields [19]. As the intrinsic noise of MTJs is higher than in SV, the former only has a better performance due to higher SNR.

#### 2.1.1. Noise Sources in MR Sensors

To measure a magnetic signal of interest, its amplitude must be substantially higher than the devices’ noise level. The noise of a MR sensor comes mainly from magnetic fluctuations associated to magnetic domain nucleation and displacement within the sensing layer. The main noise components in a MR sensor are: thermal (electronic) and shot noise contributions (being the latter only present in devices with tunneling mechanism), thermal magnetic noise, random telegraph noise (RTN) and 1/*f* (electronic and magnetic) noise [20,21,22].

Thermal (electronic) noise [23] arises from the random thermal motion of electrons responsible for collisions with impurities and other electrons, presenting therefore a dependence on the device resistance (*R*) and absolute temperature (*T*), described by the Nyquist formulation:

(1) $$S_{V}^{th}\left[V^{2}/\text{Hz}\right]=4k_{B}TR$$

where *k_B_* stands for the Boltzmann constant. This component only vanishes at absolute zero, and shows a flat behavior along the entire frequency band. Once the thermal velocity of electrons is higher than its velocity in the conducting media, $S_{V}^{th}$ has no dependence on the bias current. Shot noise [24] is caused by circuit discontinuities, in this case the insulating layer in MTJs resulting from the charge carrier’s discrete nature, originating a pulsed current and thus exhibiting fluctuations in a short time scale. This fluctuation power density increases with the device bias current (I) and is independent of the frequency:

(2) $$S_{V}^{sh}\left[V^{2}/\text{Hz}\right]=2qIR^{2}$$

where *q* is the electron charge. Shot noise is not present in SV sensor devices.

As the dimensions of a MR sensor are decreased, random fluctuations of the sensing layer magnetization occur under thermal excitation, becoming comparable with the thermal (electronic) noise. These thermally activated fluctuations are the source of thermal magnetic noise, which is frequency independent and inversely proportional to the sensing layer volume [20,25,26].

The random magnetization fluctuations in the sensing layer caused either by a repeated capture of electrons into trapping centers [27] or by the displacement of domain walls [20,28] are the origin of RTN (or Barkhausen effect), which exhibits a Lorentzian type frequency behavior. RTN in not always evident, being shadowed in the low-frequency band by the 1/*f* magnetic noise.

The 1/*f* noise, composed of an electronic and magnetic component, has a spectral density inversely proportional to the frequency, being the dominant source in the low-frequency regime [20,27]. The electronic part (1/*f* electronic) arises from voltage fluctuations related to charge trapping in crystal defects. The oscillations in the sensing layer magnetization caused by domain wall pinning and depinning at defect sites is behind the origin of the magnetic part (1/*f* magnetic) [22]. Therefore, the maximum density of 1/*f* magnetic noise occurs in the linear transition of the sensor, where the magnetization of the sensing layer is switching between the two saturation states. The 1/*f* magnetic noise is dominant in the sensor linear range and is mostly suppressed in the saturation states [29]. The 1/*f* spectral density is empirically described by the Hooge formulation [30]:

(3) $$S_{V}^{1/f\left(SV\right)}\left[V^{2}/\text{Hz}\right]=\frac{\gamma_{H}}{N_{c}}\;\frac{V^{2}}{f}$$

(4) $$S_{V}^{1/f\left(MTJ\right)}\left[V^{2}/\text{Hz}\right]=\frac{\alpha_{H}}{A}\;\frac{V^{2}}{f}$$

where $\gamma_{H}$ ($\alpha_{H}$) is the (modified) Hooge constant, $N_{c}$ is the number of charge carriers (proportional to pillar area A in a MTJ), *f* the operating frequency and *V* the bias voltage. In standard MgO-MTJ $\alpha_{H}$ typical values are in the 10^−9^–10^−7^ µm^2^ range [17,31]. For MgO-MTJ structures with a soft pinned sensing layer this parameter increases, targeting the 10^−7^–10^−5^ µm^2^ range indicating higher intrinsic noise [17,32,33,34]. Above the 1/*f* knee, thermal and shot noise become predominant, overlapping the 1/*f* spectral density.

The magnetic noise of a MR sensor (comprising both 1/*f* and thermal magnetic components) shows a linear dependence on the sensitivity [21,26]:

(5) $$S_{V}^{mag}\left[V^{2}/\text{Hz}\right]=\beta \frac{k_{B}TV^{2}}{\pi \mu_{0}M_{s}\Omega f}\frac{∆R}{R}\left(\frac{1}{R}\frac{dR}{dH}\right)$$

where Ω and *M_S_* are respectively the volume and the saturation magnetization of the sensing layer, μ_0_ the magnetic permeability of free space and β is the ratio between the imaginary and real parts of the sensing layer transverse magnetic susceptibility. Equation (5) is deduced using the fluctuation dissipation relation considering the system in thermal equilibrium [21].

At high-frequency only thermal noise source is present for both SVs and MTJs. Notice that this level is now 2–3 orders of magnitude lower than in low-frequency regime.

**Figure 2:**
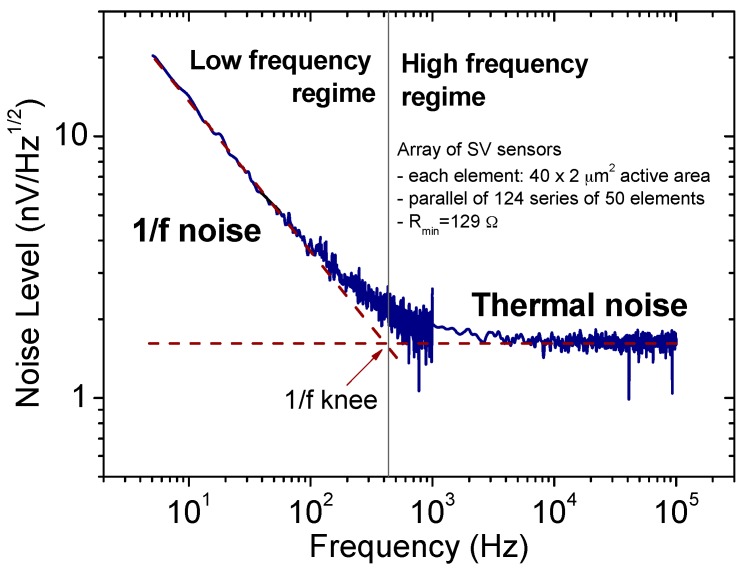
Typical noise spectrum of a MR device (SV array), reaching the thermal level for frequencies around 400 Hz. In the low frequency regime the 1/*f* noise is dominant, while in the high frequency regime the spectrum is reduced to its thermal level. Both electronic and magnetic noise components are present since the spectrum was recorded with the sensor operating on its linear range.

Figure 2 shows a representative curve for the noise of a MR sensor (DC to 100 kHz), where the transition from 1/*f* domain to the thermal level is clearly observed. One can thus, identify the optimum operation regime with a minimum noise above the 1/*f* transition. This is not compatible with the detection of low frequency fields, such as for biometric signals or space applications.

#### 2.1.2. Detection Level Enhancement of MR Sensors

The minimum detectable field at a specific frequency depends on the sensor’s intrinsic magnetic noise (targeted lowest possible) and its sensitivity (*dR/dH*). On the one hand, particular noise reduction strategies act mainly at the low-frequency range leaving the high-frequency noise almost unchanged. On the other hand, an improvement of the sensor sensitivity also results in an overall detection level enhancement.

For applications that do not require a high spatial resolution, the use of a large array of N elements connected in series is a reliable alternative to improve the detection limit, since the SNR improves with N^1/2^, although the noise level increases by a factor N [11,35]. A major disadvantage of this approach is the increase in total device resistance, which not only implies a higher thermal noise background but also leads to a more complex integration with electronics. To overcome this problem, the use of a parallel of elements in series instead of a simple series yields a resistance reduction [36]. In addition, patterning a single sensing element with a large area (higher number of carriers) contributes directly for the noise level reduction at low frequency [10,12,18]. However, large patterned ferromagnetic materials display magnetic domains, hence introducing RTN in the magnetic response.

The integration of MFCs is the used strategy to enhance significantly the sensor sensitivity. These elements increase the magnetic flux through the sensor and consequently decrease the linear operating range without introducing additional noise [10,12,31,37,38]. Depending on the geometry and profile, MFCs can yield a sensitivity gain up to 100 times [39], reflecting in a detection level decrease. A brief overview on MFCs integration is presented in Section 2.3.

Table 1 shows typical values of detection level obtained for a single SV sensor (active area: 40 × 2 µm^2^) at 30 Hz, where 1/*f* is dominant; and at 10 kHz, in the thermal regime. These are compared with sensor in the gap of funnel shaped MFCs (gain ~10, gap = 4 µm) and an array of 992 SV elements connected in parallel of series. A bias current of 1 mA was used in each device.

Increasing the bias current improves the detection level in the high frequency range [32], remaining unchanged below the 1/*f* knee. Therefore, the use of MR sensors with large current biasing can be faced as a viable alternative for applications requiring an improved detectivity at high frequencies.

Table 1 clearly shows that the detection in the low-frequency regime is limited. A sophisticated way to overcome this low-frequency restriction consists in the integration of MEMS with MFCs and MR sensors, whose motion modulates the interest signal and displaces it to the thermal regime [40,41]. This hybrid strategy is discussed in next sections.

### 2.2. MEMS Resonators

Microelectromechanical systems (MEMS) resonators have a wide range of applications in transducing technologies [42], either sensing devices with great demand on pressure sensors [43,44] and accelerometers/gyroscopes [45,46], or micro-actuators, taking advantage of a high frequency operation and low power consumption. More recently, MEMS resonating technology has been integrated in more mature sensing applications, as magnetometers based on Lorentzian-force-generated mechanical resonance [47,48], replacing quartz crystal oscillators for signal process [49], or biosensors for label free differentiation of bio-molecules [50,51,52].

The most common structures used as MEMS resonators are: (i) suspended cantilevers and (ii) torsional bridges, which vibrate at high frequencies (MHz range) when actuated by a gate voltage. MEMS torsional paddle bridges-torsional vibrating mode-present higher deflection amplitudes than cantilevers-flexural vibrating mode. The maximum oscillation amplitude corresponding to the most sensitive operation point, occurs at its resonance frequency, and is expressed by:

(6) $$f_{0}\left[\text{Hz}\right]=\frac{1}{2\pi }\;\sqrt{\frac{K}{m}}$$

where *m* is total resonator mass and *K* corresponds to the spring constant (dependent on the resonator dimensions and mechanical properties). A common technique to evaluate the MEMS deflection is measuring the electrical capacitance between the resonator and the gate electrode. In addition, MEMS can behave as actuators since a voltage (both DC and AC) between the resonator and the gate generates an electrostatic force between these two structures (capacitor plates), inducing an oscillation in the former.

**Figure 3:**
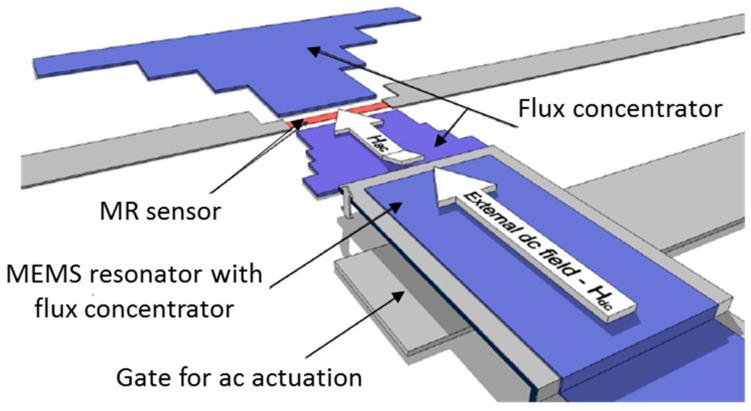
Schematic of a MR-MEMS hybrid device used for high frequency modulation of (quasi-)DC magnetic fields, where a MEMS cantilever with incorporated MFCs oscillates at high frequency to modulate the signal of interest. (Courtesy of Guedes A.).

The combination of robustness and high sensitivity of MR sensors with the low power consumption and high frequency operation of MEMS resonators results in high frequency magnetic field modulation, deflection detection of micro-bridges [53], and 1/*f* noise suppression in SVs and MTJs. Microelectromechanical flux concentrator, where MFCs are defined on MEMS flaps can then be integrated with MR sensors. The novelty of this device relies on the mechanical modulation of (quasi-)DC fields at the sensor position achieved by the MFCs oscillation at a frequency corresponding to the MEMS resonance, allowing a shift in the operating point to the high-frequency range [40,41,54,55,56,57,58,59,60]. A schematic is shown in Figure 3.

A different application of a MR-MEMS hybrid device was developed at INESC-MN [61]. In this device, a CoCrPt permanent magnet was defined on top of a cantilever while a SV element was fabricated near the cantilever’s free end. Such device showed to be capable of detecting the cantilever’s mechanical deflection. The field sensed by the SV changes according to the permanent magnet position induced by the deflection. This hybrid device displayed a resolution of 0.06 Å/Hz^1/2^.

An alternative to the described mechanical field modulation consists in the use of chopping techniques to modulate the sensitivity of MR sensors and consequently overcome the 1/*f* noise [62]. A parallel chopping approach demonstrated a slight reduction of noise spectrum at very low frequency values.

### 2.3. Magnetic Flux Concentrators

**Figure 4:**
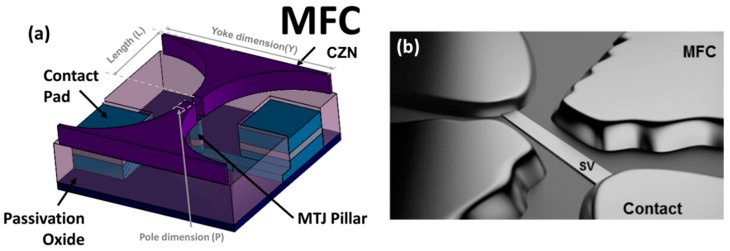
(**a**) Schematic view of MFCs with funnel shape geometry and respective integration with a patterned MTJ sensor (before the top contact deposition). Reprinted with kind permission of The European Physical Journal (EPJ); (**b**) Illustration of steep profile MFCs close to a SV single sensor. Reprinted with permission from Leitao D.C. *et al.*, *Sensors* 2015, *15*, 30311–30318. Copyright 2015 MDPI.

The integration of magnetic flux concentrators (MFCs) is a reliable strategy to enhance significantly sensor’s sensitivity when the device footprint is not an issue for the application. Being made of soft ferromagnetic materials (e.g., NiFe [63] or amorphous Co based alloys [64]) and with an appropriate geometry, MFCs concentrate the external field in the region where the sensor is placed. The effective gain introduced by the MFCs is defined by the ratio between the magnetic field in the gap and external field. The gain depends on the geometrical parameters (e.g., length, pole distance, yoke/pole ratio—Figure 4a) [10,17,32,65] and intrinsic magnetic properties of the material (e.g., magnetic permeability). Thereby, upon MFCs geometry optimization and material control, a maximum enhancement of the field detection can be achieved. Figure 4b illustrates the integration of MFCs with a SV sensor, with focus on the concentrators’ gap region where the sensing element is placed.

The profile of the MFCs also influences the gain of the device. MFCs patterned with a funnel shape and a steep profile (90°, defined by lift-off) in the gap region provided a sensitivity enhancement up to 30 times [12,17,32]. MFCs made of the same magnetic material and with the same pole-sensor distance, but with a 3D tapered profile at the pole region (45°, defined by ion milling etch), lead to an increased magnetic flux concentration resulting in sensitivity gains up to 100 times [40].

**Figure 5:**
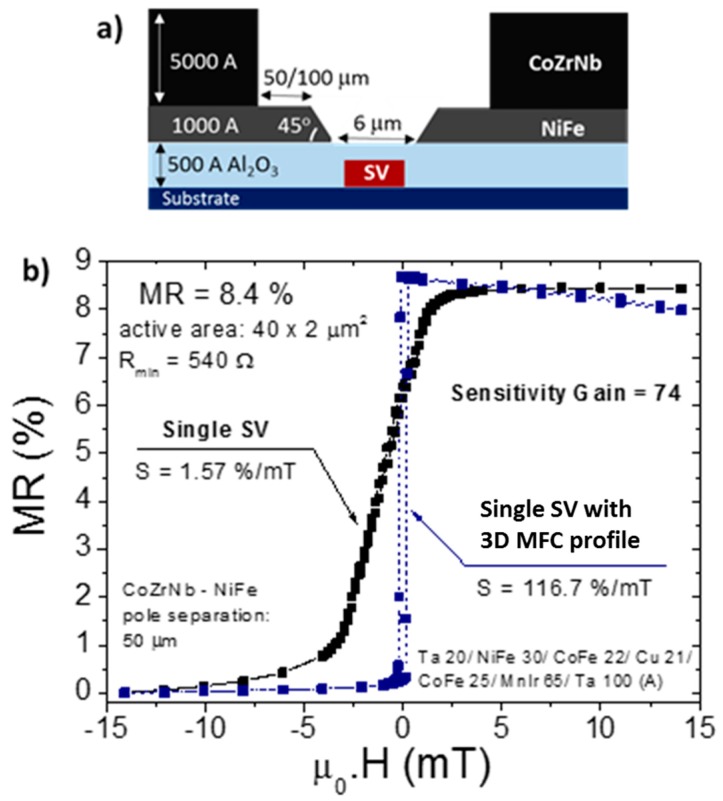
(**a**) Schematic cross section of the double layer MFC with a 3D tapered profile, showing a pole angle of 45° in the NiFe film and a steep profile in the CoZrNb pole, integrated with a SV sensor; (**b**) Effect of the 3D tapered profile MFC integration on the magnetotransport curve of a single SV sensor, showing a sensitivity enhancement of 74 times.

However, an important characteristic of this single layer MFC-MR sensor integration approach, common to both steep and 3D profiles, is a significant thickness difference between MFCs and sensor. This results in major losses of the concentrated flux since it is not captured by the sensing device. To overcome this particularity and guide a larger amount of magnetic flux to the sensor, a more complex structure combining steep and tapered profiles was developed. This new approach is shown in Figure 5a and consists in a double layer MFCs: starting with a thin layer of Ni_80_Fe_20_ (0.1 µm thick, profile angle 45°) in the pole region; and a second layer of CoZrNb (0.5 µm thick, steep profile). The CoZrNb pole is separated by 50–100 µm from the Ni_80_Fe_20_ pole, reducing considerably the MFCs thickness at the pole region. With this configuration, the field kept by the entire structure is driven into the thin Ni_80_Fe_20_ layer, minimizing the flux concentration losses in the sensing region, when compared with traditional single layer MFCs (Figure 5b).

### 2.4. Hybrid Technology Integrating MR Sensors, MEMS and MFCs

The MEMS flux concentrator integrated with MR sensors is a reliable solution to overcome the problem imposed by the 1/f noise level in the detection of magnetic signals in low-frequency regime. Edelstein *et al.* proposed a design [40,41] consisting of a SV sensor defined between two MFCs (2 × [Cr 40 Å/Ni_80_Fe_20_ 1500 Å]) with trapezoidal shape and deposited on silicon MEMS flaps (MFCs gap = 52 µm), presenting normal-mode resonant frequencies in the 10 kHz range. The oscillatory movement of the MEMS flaps is induced by electrostatic comb drives or by applying an AC voltage between the flap and a gate electrode. This modulates the field in the sensing area shifting the sensor operation point to high frequencies. Recently, a distinct MEMS drive method using piezoelectric materials was introduced [56,58,59,60]. The output voltage of the MR sensor induced by the resonator with incorporated MFCs has a magnetic contribution arising from the modulated field. For capacitive MEMS, an electric contribution also appears caused by a coupling between the sensor and the resonator gate electrode [55,57]. While piezoelectric MEMS oscillates at the same frequency of the driving signal (*f*), the components of the driving signal influence the motion of capacitive MEMS. For the latter, a pure AC voltage at frequency *f* causes a vibration at twice the frequency (2*f*) [53], while an AC voltage with a DC component originates a motion at *f* (*f* and 2*f*) if the voltage offset is higher (lower) than the alternating voltage amplitude.

Depending on the spatial arrangement (e.g., in-plane, over the sensor), the field can be modulated at the oscillating frequency or at twice this value. Considering that the MEMS flaps are in the plane of the sensing element, two cycles of magnetic field (minimum field/maximum field/minimum field) occur in each cantilever mechanical cycle (out-of-plane up/in-plane/out-of-plane down). Therefore, when the MEMS flaps are driven to oscillate at their resonance frequency (*f_0_*), a static magnetic field is modulated at 2*f*_0_—output frequency. However, when a quasi-DC magnetic field (frequency *f_m_*) is the signal of interest, the field modulated at 2*f*_0_ is in fact amplitude modulated [56]. In this case, considering the modulation waveform field *B_m_*(*t*) and the carrier waveform *B_c_*(*t*):

(7) $$B_{m}\left(t\right)=M\cos\left[2\pi f_{m}t\right]$$

(8) $$B_{c}\left(t\right)=A\cos\left[2\pi \left(2f_{0}\right)t\right]$$

where *M* and *A* are the amplitudes of the modulation (*i.e.*, low-frequency field accounting already for the gain factor introduced by the MFCs) and carrier wave (originated by the MEMS resonance), respectively. The magnetic field reaching the sensor (*B_s_*(*t*)) results from the amplitude modulation of these signals [66,67], obtained from multiplying the carrier waveform by the quantity [1 + *B_m_*(*t*)]:

(9) $$\begin{array}{c} B_{s}\left(t\right)=[1+M\cos\left[2\pi f_{m}t\right]]\cdot A\cos\left[2\pi \left(2f_{0}\right)t\right]\\ =A\cos\left[2\pi \left(2f_{0}\right)t\right]+\frac{1}{2}A\cdot M\left\{\cos\left[2\pi \left(2f_{0}-f_{m}\right)t\right]+\cos\left[2\pi \left(2f_{0}+f_{m}\right)t\right]\right\} \end{array}$$

Equation (9) shows that *B_s_(t)* (already modulated) encloses three components: the carrier waveform *B_c_(t)* which remains unchanged, and two sidebands at frequencies *2f_0_* ± *f_m_*. Therefore, it is possible to detect the low frequency magnetic field (*f_m_*) in the high-frequency regime (*2f_0_*) by reading the output voltage of the sidebands *2f_0_ − f_m_* and *2f_0_ + f_m_*, which can be easy understood calculating the Fourier transform of *B_s_(t)* [66,67]:

(10) $$ℱ\left(B_{s}\right)=A\delta \left(2f_{0}\right)+\frac{1}{2}A\cdot M\delta \left(2f_{0}-f_{m}\right)+\frac{1}{2}A\cdot M\delta \left(2f_{0}+f_{m}\right)$$

showing a delta-Dirac function at *2f_0_* and at the two sidebands frequencies.

However, when the MEMS flaps are over the sensor element (not in the same plane) [55,58,59,60], one cantilever mechanical cycle corresponds to a single magnetic field cycle. Therefore, with this geometry, the modulation of a static magnetic field occurs at the same frequency of the MEMS periodic motion (*f*_0_). Similar calculations replacing Equation (8) by *B_c_(t) = A*cos[*2*π*f_0_t*] indicate that a quasi-DC signal can be recovered from the sidebands *f_0_ ± f_m_* in the modulation process.

The MEMS modulation efficiency (*e*_MEMS_) is proportional to the cantilever’s mechanical displacement, increasing for larger deflection amplitudes. *e*_MEMS_ is defined as the ratio between the amplitudes of the modulation and carrier waveforms [56], translating the carrier amplitude variation relative to its unmodulated level. A maximum efficiency *e*_MEMS_ = 100%, corresponds to a maximum amplitude change, representing a full amplitude modulation and the highest possible SNR. In practice, several factors may affect the device performance, thus reducing *e*_MEMS_. One design/fabrication feature, which directly affects *e*_MEMS_ is the distance between MFCs and MR sensor, which must be the smallest possible to ensure a better modulation efficiency.

Other important parameter to characterize the MEMS resonators is the quality factor (*Q*) [68], which quantifies how under-damped is the oscillating motion and characterizes its frequency bandwidth. A high *Q* is associated to large amplitude stable deflections with an accurate oscillation frequency, indicating small energy dissipation. The friction induced by the physical medium where the resonator operates is the main limiting factor of *Q*. A vacuum packaging is a reliable alternative to minimize the resonator damping motion [40,69].

## 3. Towards picoTesla Field Detection

Although the use of hybrid MEMS flux concentrators devices with integrated MR sensors seems to be a promising technique for the detection of ultra-low intensity magnetic fields (pT/Hz^1/2^ range), this strategy (tens of kHz) has been validated only by few research groups [40,41,54,55,56,57,58,59,60]. The successful merging of MR and MEMS technology represents a great technical challenge. The combination of state-of-the-art high sensitive and low-noise MR sensors with the required steps to micro-fabricate and release the MEMS structures reveals to be a critical aspect in the development of these hybrid devices and is a limiting factor for the efficiency of this modulation technique. An overview of the results reported by five different groups is discussed in the following focusing on key points as geometries and operating conditions culminating with achieved magnetic field detection. Then on Section 3.5 the fabrication challenges for these devices are discussed.

### 3.1. Approach I: MR Sensors, Coupled Capacitive MEMS with MFCs

**Figure 6:**
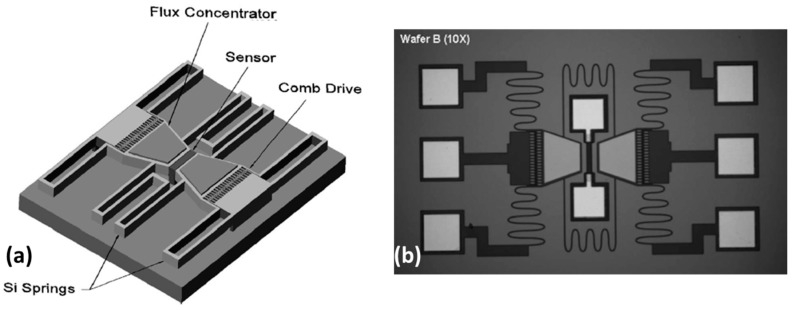
(**a**) Schematic view and (**b**) SEM image of the proposed MEMS flux concentrator device. Reprinted with permission from Edelstein A.S. *et al.*, *J. Appl. Phys.* 2006, *99*, 08B317. Copyright 2006 AIP Publishing LLC.

The MEMS flux concentrator developed by Edelstein *et al.* [40,41] consisted of a SV sensing element (active area: 3 × 88 μm^2^) placed between two Si cantilever resonators. These were fabricated on silicon-on-insulator (SOI) wafers and incorporating trapezoidal bilayers of Cr/Ni_80_Fe_20_ [70] acting as MFCs on top. The height of the trapezoid was set in 104 μm, while the pole and yoke had a width of 80 and 150 μm, respectively, corresponding to a field amplification of 2 (MFCs gap = 52 μm). The reduced MFCs size is required to ensure that the MEMS resonates in the desired high frequency band. Due to non-uniformity in the MEMS fabrication process, a dispersion in the resonance frequency of the two cantilevers occurs. Then, besides displaying oscillations not entirely in phase, the relative phase also tends to drift. To overcome this problem, polysilicon springs (4 μm wide) connecting the two MEMS flaps were incorporated in the design, inducing a mechanical coupling which forces a common normal-mode resonance frequency. Figure 6 shows a schematic view of the developed MEMS flux concentrator design and a SEM top view of the fabricated device. With this design a 180° out of phase motion of the MEMS flaps corresponds to a higher resonance frequency (second normal resonance mode) when compared with the in-phase oscillating motion (first normal resonance mode). The resonant vibration mode is induced by electrostatic comb drives introduced in the design.

This structure is characterized by a resonance frequency *f_0_* ≈ 24 kHz and a corresponding quality factor *Q* ≈ 30 [54]. Validation was achieved by shifting magnetic signals with a frequency of 25 Hz and intensities of 22 μT and 1.3 μT to the high frequency range (around 48 kHz) and recording the respective sidebands. The resonator flaps were driven into an in-phase oscillating motion, exhibiting a maximum vertical deflection of 5 μm.

### 3.2. Approach II: MR Sensors, Capacitive MEMS (Single Cantilever and Torsional Paddle) with MFCs

To modulate a low-intensity and low-frequency magnetic field into the high frequency range, INESC-MN explored two different hybrid MEMS flux concentrator devices: (i) SV sensor with static MFCs and a single MFC-MEMS cantilever [55]; and (ii) a MTJ sensor placed under a MEMS torsional structure with both static and oscillating MFCs [57].

**Figure 7:**
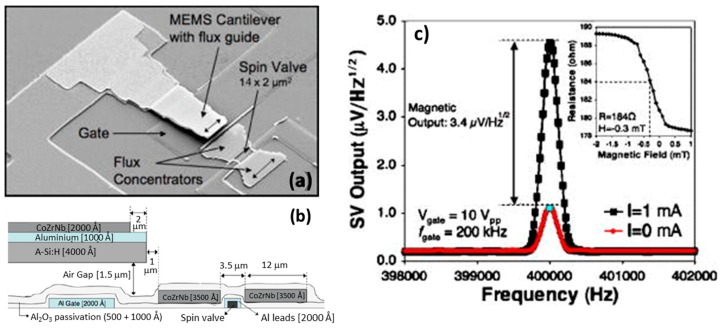
(**a**) SEM image of the hybrid device showing the integration of a SV sensor with static MFCs and a single MEMS cantilever resonator (with an additional MFC on top); (**b**) Schematic view of the device cross section; (**c**) SV voltage output exhibiting both electric (capacitive coupling) and magnetic components when the cantilever resonates at a frequency of 200 kHz. Reprinted with permission from Guedes A. *et al.*, *J. Appl. Phys*. 2008, *103*, 07B924. Copyright 2008 AIP Publishing LLC.

In the first approach, a single SV sensor (active area: 10 × 2 μm^2^) is patterned together with two static trapezoidal MFCs (350 nm thick amorphous CoZrNb alloy), presenting a gap of 3.5 μm [55]. A micro-machined MEMS cantilever resonator of hydrogenated amorphous silicon (400 nm thick, 30 × 16 μm^2^ area) with an air gap of 1.5 μm between its bottom surface and the gate electrode is fabricated as close as possible to one of static MFCs (separation of 1 μm). The gate electrode consists of a 200 nm thick Al lead placed under the cantilever structure, while a thinner Al layer (100 nm thick) is deposited on the cantilever surface to act as counter-electrode for electrostatic actuation. An additional MFC (CoZrNb 200 nm) is deposited and patterned on top of the cantilever. The characterization of this combined MFCs structures results in a field gain of 5 times. A SEM image of the fabricated device and a cross section schematic view are presented in Figure 7.

Applying an AC signal with an amplitude of 10 V_pp_ and a frequency of 200 kHz to the device gate electrode, makes the cantilever oscillate at 400 kHz, being able to modulate a DC external magnetic field of 0.3 mT. The SV sensor output (bias current: 1 mA) has an electric component coming from the direct AC coupling with the gate electrode (1.2 μV/Hz^1/2^) and a pure magnetic contribution (3.4 μV/Hz^1/2^), visible in Figure 7c. However, this particular device shows a low modulation efficiency from DC to AC (*e*_MEMS_ = 0.11%), so the improvement by operating the sensor at its thermal background is mitigated by signal losses due to low *e*_MEMS_. A minimum detectable static field of 540 nT/Hz^1/2^ is reported.

**Figure 8:**
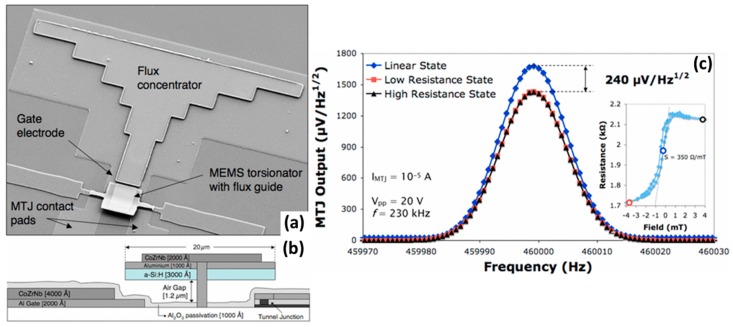
(**a**) SEM image of the device exhibiting the MEMS torsional structure integrated with a MTJ sensor and both static/dynamic MFCs; (**b**) Schematic view of the device cross section; (**c**) MTJ voltage output corresponding to the detection of a DC magnetic field of 0.36 mT when the torsional structure is actuated at a frequency of 230 kHz. Both magnetic and electric (capacitive coupling) components are presented. Reproduced with permission from Guedes A. *et al.*, *IEEE Trans. Magn.* 2008, *44*, 2554; published by IEEE, 2008.

A different approach consists that uses a MEMS torsional structure (width 20 μm) fabricated by surface micromachining with hydrogenated amorphous silicon (300 nm thick). The sensing element is now a MgO MTJ sensor (pillar area: 15 × 15 μm^2^) placed under the torsionator [57]. A single 400 nm thick CoZrNb static MFC was patterned close to the MTJ, while an additional 200 nm thick layer of this soft material was deposited and patterned on top of the MEMS torsional structure. Both gate electrode and counter electrode were defined as in the first approach. Figure 8 shows a SEM image of the fabricated hybrid device and a schematic view of its cross section. This device was used for DC magnetic field modulation and detection at high frequencies, in an attempt to improve the MEMS modulation efficiency and the minimum detectable field. Figure 8c shows the sensor voltage output when an AC signal of 20 V_pp_ and a frequency of 230 kHz is applied to the device gate electrode. The field detected by the MTJ sensor is modulated at 460 kHz as a result of the torsional bridge resonating motion. The difference between the results obtained for the sensor operating in the linear range or at saturation corresponds to the magnetic component (~240 μV/Hz^1/2^). The device’s *e*_MEMS_ (from DC to AC) is about 11%. This *e*_MEMS_ enhancement reflects the higher deflection amplitude achieved by this type of structures when compared to resonator cantilevers. For this operating condition, the DC field detection limit is 40 nT/Hz^1/2^, improving by about one order of magnitude compared to the initial situation characterized by a low *e*_MEMS_ (detection level of 540 nT/Hz^1/2^).

### 3.3. Approach III: MR Sensors, Piezoelectric MEMS with MFCs

Another successful example of such a hybrid device integrates SVs and piezoelectric MEMS cantilevers. Piezoelectric MEMS devices have a number of advantages over electrostatic MEMS presented above. They require lower driving voltage (~one order of magnitude for PZT based cantilevers [71]), are more area efficient and do not require air-gap capacitors with complex geometries and exposed conductors. The principle of operation of piezoelectric cantilevers is based on the property of the active material employed in its manufacturing-piezoelectricity. Piezoelectric materials generate an electric voltage when subjected to a mechanical deformation. One of the unique characteristics of this phenomenon is its reversibility; materials exhibiting the direct effect of piezoelectricity will also experience the inverse effect, a mechanical deformation when an electric field is applied. One drawback of piezoelectric MEMS is the more complex fabrication process when compared with capacitive MEMS. However, from a performance point of view, piezoelectric MEMS are more attractive [71].

**Figure 9:**
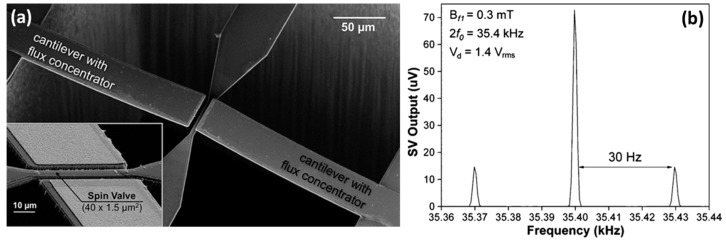
(**a**) SEM image of the developed device, showing the SV sensing element between two resonator cantilevers with integrated MFCs; (**b**) Acquired data resulting from the modulation of a low-frequency AC field (0.3 mT with a frequency of 30 Hz), exhibiting the respective sidebands when the cantilevers are actuated at the resonance frequency (*f*_0_ = 17.7 kHz). Reproduced with permission from Guedes A. *et al.*, *IEEE Trans. Magn.* 2012, *48*, 4115; published by IEEE, 2012.

Although employing a different driving mechanism for the MEMS cantilevers, this approach has a design similar to the one proposed by Edelstein *et al* [40,41]. Single SV element (active area: 40 × 1.5 μm^2^) is placed in the gap of two SOI/Mo (100 nm)/AlN (700 nm) piezoelectric MEMS cantilevers with patterned MFCs on top [56]. Figure 9a displays a SEM picture of the fabricated hybrid device, depicting the SV sensor suspended on a bridge centered in the gap between the two AlN-based piezoelectric cantilevers. In this case, multilayers of antiferromagnetic coupled CoFeB layers were used as MFCs ([CoFeB 38 Å/Ru 18 Å] _× 32_/CoFeB 38 Å). The cantilever resonator structures were patterned with a fixed width of 40 μm, and different lengths ranging from 200 to 400 μm. The larger cantilever structure has a resonance frequency *f*_0_ = 17.7 kHz, a quality factor *Q* = 181, and *e*_MEMS_ = 1.6%. Figure 9b shows the sidebands in the high frequency range obtained from the modulation of an 0.3 mT external magnetic field with a frequency *f_m_* = 30 Hz, upon a cantilever actuation with 1.4 V_rms_ at *f*_0_. The minimum detectable field of the device is 301 nT/Hz^1/2^ for the detection of DC magnetic fields, and 602 nT/Hz^1/2^ for low frequency fields (taking into account the ½ loss from the modulated high-frequency sidebands).

**Figure 10:**
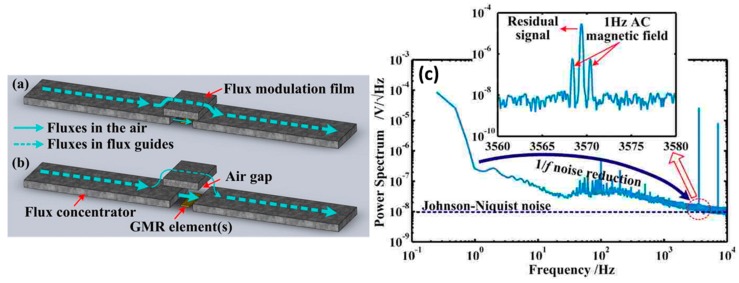
Schematic operating view of the VMFM based device: (**a**) cantilever gets close the sensing elements, reducing the magnetic flux through them; (**b**) cantilever moves upwards, restoring the magnetic flux in the sensing elements; (**c**) Acquired device output resulting from the modulation of a low-frequency AC field (1.2 μT with a frequency of 1 Hz), exhibiting the respective sidebands when the cantilevers are actuated at the resonance frequency (*f*_0_ = 3.57 kHz). The residual signal at *f*_0_ arises from the remanence of the Ni_79_Fe_21_ flux modulation field and/or from an electric coupling between then sensing elements and the MEMS piezoelectric structure. Reprinted with permission from Hu J. *et al.*, *Appl. Phys. Lett.* 2012, *100*, 244102. Copyright 2012 AIP Publishing LLC.

A parallel approach based on vertical motion flux modulation (VMFM) was also targeting *e*_MEMS_ enhancement [58,59,60]. In this case, a micron size piezoelectric Si cantilever is suspended above the gap between a pair of MFCs. Commercial giant-magnetoresistive (GMR) sensors in a bridge configuration are used and placed between the MFCs (gap = 60 μm). The MEMS structure is composed of a Si cantilever with a piezoelectric ceramic (lead zirconium titanate-PZT, top surface) and a soft magnetic film (bottom surface). The latter is an electroplated Ni_79_Fe_21_ layer with 8 μm thick and dimensions of 600 μm × 120 μm, being suspended at a static height of about 10 μm. These dimensions ensure that it covers the MFCs gap and thus acts as a flux modulation film as it is driven vertically by the MEMS actuator. When the cantilever approaches the MFCs gap, the magnetic flux goes preferentially through the NiFe film, decreasing the magnetic field that reaches the GMR elements. On the other hand, when the cantilever moves upwards the magnetic field in the gap region is restored. Figure 10a,b show a schematic operating view of the device. Its validation was achieved upon the modulation of a 1.2 μT AC magnetic field with a frequency of 1 Hz. The obtained output power spectrum is shown in Figure 10c, where the two high frequency sidebands are observed. The cantilever structure oscillates under a resonance frequency *f*_0_ = 3.57 kHz, reaching a displacement amplitude of 8 μm (in the air) under an AC driving voltage (6 V) which corresponds to a *Q* ≈ 14. The *e*_MEMS_ of this device is about 19%. The shift in the sensor operating point to the high frequency range causes a noise level reduction around 18×, corresponding to a DC minimum detectable filed of 530 pT/Hz^1/2^.

### 3.4. Approach IV: MR Sensors, Bulk-Micromachined MEMS with MFCs

A final example resorts to in-plane, bulk-micromachined electrostatic actuators combined with SV sensors and MFCs. The modulation of the magnetic field sensed by the SVs is achieved by having part of the MFC patterned atop a large stroke micro actuator. Such actuators are obtained by standard micromachining, deep reactive ion etching (DRIE) of silicon-on-insulator (SOI) substrates [72]. When compared to other types of MEMS devices (e.g., surface micromachined structures [53,73]), larger displacements can be achieved by a simple design of the movable and anchor parts, dimensioning of the inertial mass, parallel flexures and interdigitated comb drives [74]. Larger modulation effects can therefore be expected, at the expense of lower resonance frequencies [74,75].

**Figure 11:**
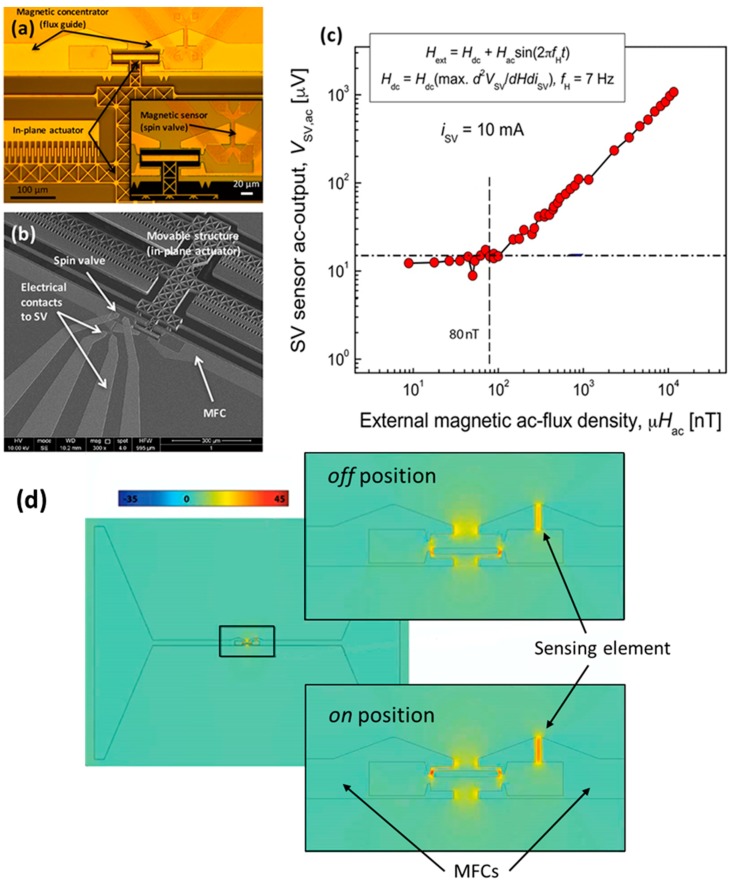
(**a**) Optical and (**b**) SEM image of modulator combining in-plane electrostatic microactuators with interdigitated fingers comb drive, SV sensors and MFCs; (**c**) Modulated sensor output as a function of magnetic field amplitude at low frequency (7 Hz); (**d**) Finite element simulation which illustrates the concentrated magnetic flux in the sensing element for the two positions: off-minimum concentration; on-maximum concentration.

Figure 11a,b shows images of a fabricated device with SV sensors (active area: 2 × 20 μm^2^) and MFCs of 400 nm thick CoZrNb [57]. The MEMS are actuated applying an AC voltage with DC component to the comb drive (electrostatic driving), responsible for a motion at *f*_0_ and 2*f*_0_. The actuator has *f*_0_ = 7.5 kHz and vibration amplitude of 17.5 µm. Despite such large displacement, it only reaches an efficiency *e*_MEMS_ = 1.03%, comparable to that of the modulator described in [56]. This low *e*_MEMS_ value is attributed to the larger gaps used in the bulk micromachined device. The detectivity is around 80 nT/Hz^1/2^ for quasi-DC magnetic fields (7 Hz), as can be extracted from Figure 11c. A simulation of the device illustrating the magnetic flux concentration in the sensing elements is presented in Figure 11d, highlighting the minimum (off) and maximum (on) concentration positions. Current work to increase the efficiency of these devices focus on further increasing the actuators displacements above 100 µm, reducing the separation between MFCs and SVs, optimizing the concentration on/off (*i.e.*, design layout and improved materials) and increasing their resonance frequency targeting a reduction of the inertial mass alongside an increase of the spring constants but without compromising displacement.

### 3.5. Fabrication Details on MEMS Integration with MR Sensors

The exchange bias in the AFM/FM interface of MR sensors is set by magnetic annealing. Consequently, the thermal stability of the sensing elements must be taken into account during its fabrication process (e.g., ion milling etch, metallization, passivation), ensuring a working temperature <150°. The standard fabrication process of the described hybrid technologies starts with the microfabrication of MR sensors, being followed by the definition of the MEMS structures. Therefore, after being patterned the sensing elements are subjected to conditions, which can affect both their magnetic and physical properties. However, these conditions (e.g., high temperature, presence of high reactive chemical etchants) are required for the MEMS fabrication, which comprises its geometry definition, electrodes deposition and structure release.

A common technique to remove the sacrificial layer (typically SiO_2_) holding the resonator consists in the use of vapor HF [76]. The major drawback of this technique is the consequent physical damage/corrosion of the MR structure. To avoid the presence of such reactive solution, oxygen plasma can be used as an alternative removal method. The latter does not affect the physical integrity of the sensing element, however the required high temperatures are an issue not only for the sensor magnetic response but also for the magnetic behavior of the MFCs materials. A final annealing step to recover the sensor magnetic response is not a viable solution since the magnetic properties of the MFCs would not be recovered, compromising the obtained flux gain.

In the approaches using SOI wafers, a backside micromachining of the wafer is required to remove the bottom Si (thickness of few μm) until the sacrificial layer. However, due to the large aspect ratio of the intended cavities, the DRIE on SOI wafers leads to silicon grass formation [77] and consequently changes the MEMS physical properties (e.g., heavier resonator with different *f*_0_). To overcome this problem, a wafer backside grinding can be performed reducing the cavities aspect ratio.

## 4. Final Remarks

This paper reviews the performance of hybrid devices resulting from the integration of MR sensors with MEMS resonators, in an attempt to detect DC or low-frequency external magnetic fields through their mechanical modulation. The latter consequently shifts the signal to the high frequency range due to the incorporation of MFCs on MEMS. This merging technology allows the MR sensor to operate at high frequencies, overcoming the higher noise spectrum density present at low frequencies as a result of the 1/*f* component.

Table 2 summarizes the results obtained for the performance of the different MEMS flux concentrator strategies presented in this review, highlighting the minimum detectable field, operation frequency and modulation efficiency. The integration of a MTJ sensor with the MEMS torsional structure [57] proved to be a reliable alternative, allowing an enhancement of static DC field detection limit from hundreds to tens of nT/Hz^1/2^, mainly due to the increase in the modulation efficiency to 11%. The larger mechanical deflections of MEMS torsional structures and the use of a sensing element with higher signal can be on the base of the observed improvement. Furthermore, the suspension of a piezoelectric MEMS above GMR sensors placed in the gap of two static MFCs [58,59,60] provided an enhancement of the minimum detectable field to hundreds of pT/Hz^1/2^, alongside with a modulation efficiency of 19%. However, single MTJ sensors with incorporated static MFCs (no MEMS) demonstrated a better performance at low-frequencies (~100 Hz), where minimum detectable fields around tens of pT/Hz^1/2^ were reported [10,12]. The lower detection levels obtained for non-integrated MR sensors combined with its simpler fabrication process make them a more reliable alternative than hybrid MR-MEMS in terms of performance and fabrication.

The presented results underline the great potential of MR-MEMS hybrid devices in the detection of low-intensity and low-frequency external magnetic fields with a wide room for improvement. However, the application of the described devices for this purpose is limited by two main factors: (i) the MR sensor’s sensitivity and (ii) the effective 1/*f* noise suppression. The latter is achieved by the field modulation employed by the MEMS structures and quantified by the modulation efficiency factor (*e*_MEMS_). An insightful understanding of the aspects limiting the overall performance of the aforementioned hybrid devices is crucial in the development of an optimized strategy that would pave the way towards the detection of picoTesla. In terms of sensitivity, a relevant improvement is obtained with the inclusion of MFCs as shown in Section 2.3, where major optimizations on this front have been done with proven results. In addition, although MR sensors based on MTJs have a higher noise level compared with SV elements, their higher SNR leads to an enhanced detection limit, justifying the choice of MTJs when targeting picoTesla detection despite the extra complexity of the fabrication process. Regarding the drive mechanisms of MEMS structures, the approaches based on capacitive MEMS [55,57] reported the disruption of some of the MR sensors caused by the gate electrode high voltage, leading to low yield of final devices. In this scope, piezoelectric MEMS cantilevers appear as a strong alternative. As *e*_MEMS_ is directly related with the amount of external field that MEMS flux concentrators are capable of capture and thus modulate, a key aspect to improve it consists in the minimization of the distance between MEMS and sensing element. In this context, the geometry based on a pair of cantilevers [56] reveals as a promising approach, since the magnetic field is captured from both sides of the MR sensor. Since the sensor output varies accordingly with the MEMS oscillating motion, the mechanical displacement of the MEMS structure is also intrinsically related with *e*_MEMS_, achieving higher modulation values for larger deflection amplitudes. Taking this into account, the geometry and dimensions of the MEMS structures must be carefully chosen aiming a larger deflection capability. Long and narrow cantilevers can achieve large displacements at the expense of an increased device footprint. Envisaging applications requiring a small device footprint, the use of materials with different mechanical properties must be address to achieve larger deflections with small dimension resonators.

The optimization of *e*_MEMS_ to values ~50% by using MEMS structures characterized by larger deflection amplitudes together with a MFCs gain improvement arising from a tapered profile (~100×) may allow the fabricated hybrid devices to reach detection levels in the pT/Hz^1/2^ range, placing these devices at the forefront of low-intensity and low-frequency magnetic fields detection.

## Figures and Tables

**Table 1:** Comparison of typical detection levels of a single SV element, obtained in low (10 Hz) and high (10 kHz) frequency range, with the obtained results upon the inclusion of MFCs and the patterning of a large array of series connected in parallel of series.

Sensor Type	Individual Sensor Area (μm^2^)	Device Footprint (μm^2^)	Noise Level at 30 Hz (nV/Hz^1/2^)	Noise Level at 10 kHz (nV/Hz^1/2^)	Detectivity at 30 Hz (nT/Hz^1/2^)	Detectivity at 10 kHz (nT/Hz^1/2^)
Single SV sensor	40 × 2	40 × 2	84.7	4.1	21.2	1.32
Single SV sensor with MFCs	40 × 2	400 × 293	84.7	4.1	1.9	0.12
Array of 992 SV elements	40 × 2	886 × 895	2.4	1.3	2.2	0.61

**Table 2:** Comparison of the different proposed hybrid devices integrating MR sensors with MEMS flux concentrators regarding the modulation efficiency and minimum detectable field.

Used Strategy	MR Sensor	Modulation Frequency (kHz)	Modulation Efficiency (%)	Minimum Detectable Field (nT/Hz^1/2^)
INESC-MN #1: capacitive single cantilever [55]	SV	400	0.11	540 (DC)
INESC-MN #2: capacitive micro-paddle [57]	MTJ	460	11	40 (DC)
Picosense/INESC-MN: piezoelectric paired cantilevers [56]	SV	35.4	1.6	301 (DC)
				602 (AC)
Hu J. *et al.*: single piezoelectric cantilever [58,59,60]	SV	3.57	19	0.530 (DC)
INL/INESC-MN: bulk micromachined electrostatic actuator	SV	7.5	1.03	80 (AC)
		15		
